# Radiation induced temporal lobe necrosis in nasopharyngeal cancer patients after radical external beam radiotherapy

**DOI:** 10.1186/s13014-020-01560-0

**Published:** 2020-05-15

**Authors:** Vincent W. C. Wu, Shing-yau Tam

**Affiliations:** Department of Health Technology & Informatics, Hong Kong Polytechnic University, Hung Hom, Hong Kong

**Keywords:** Temporal lobe necrosis, Nasopharyngeal carcinoma, Radiotherapy, Radiation induced complication, Diagnosis and treatment

## Abstract

Radiation-induced temporal lobe necrosis (TLN) is one of the late post-radiotherapy complications in nasopharyngeal cancer (NPC) patients. Since NPC is common to have skull base infiltration, irradiation of the temporal lobes is inevitable despite the use of the more advanced intensity-modulated radiotherapy (IMRT). Moreover, the diagnosis and treatment of TLN remain challenging. In this review, we discuss the diagnosis of TLN with conventional and advanced imaging modalities, onset and predictive parameters of TLN development, the impact of IMRT on TLN in terms of incidence and dosimetric analyzes, and the recent advancements in the treatment of TLN.

## Background

Nasopharyngeal cancer (NPC) is a common cancer in south-east Asia [1]. Radical radiotherapy (RT) with or without chemotherapy is the primary treatment modality for NPC due to the tumor anatomical location and radiosensitivity [2]. Radiation dose of 66 to 70 Gy is required for the gross tumor volume (GTV) and 54 to 60 Gy is prescribed to the clinical target volume (CTV). Since NPC frequently exhibits infiltration to skull base, it is sometimes inevitable that the temporal lobes (TLs) are included in the radiation field with parts of the structure receiving over 60 Gy. Therefore, temporal lobe necrosis (TLN) is one of the late post-RT complications in NPC patients. Intensity-modulated radiotherapy (IMRT) has been the mainstay of RT treatment of NPC and the imaging modalities together with therapeutic options of TLN have been intensively developed in recent years. This review highlights the development of diagnostic procedures, the impact of IMRT on TLN, predictive parameters for the development of TLN and the different therapeutic options for the management of TLN.

### Symptoms and incidence

Majority of patients with TLN were asymptomatic even at late stage [3], while the clinical symptoms of other patients include epilepsy, dysphasia, seizure, cognitive decline, change in consciousness, memory impairment, dizziness and headache [3, 4]. TLN could also lead to severe and fatal symptoms including convulsion, intracranial hemorrhage and herniation [5]. The incidence of radiation induced TLN patients with NPC was dependent on the RT techniques and dosage. For NPC patients treated by conventional 2-dimensional RT, the 10-year actuarial incidence of TLN was ranged from 4.6% in a conventional dose 2.5 Gy per fraction scheme of 60 Gy to 18.6% in an accelerated dose scheme of 4.2 Gy per fraction [6]. Whereas other studies with incidence rates of 0.93–10.8% have also been reported [4, 7]. For patients treated with IMRT, the incidence of TLN was ranged from 4.6 to 8.5% [7–9].

### Diagnosis

#### Conventional imaging

Magnetic resonance imaging (MRI) and computed tomography (CT) can be used for detecting TLN. MRI has been deemed as superior than CT in terms of sensitivity and better soft tissue contrast [10]. The general characteristics of TLN in MRI included low signal in T1-weighted image and high signal inT2-weighted image with heterogeneous contrast enhancement and irregular or cystic shape lesions, while CT showed finger like or round hypodense lesions with post-contrast enhancement [10]. However, both conventional MRI and CT cannot efficiently differentiate TLN from tumor recurrence and therefore other advanced imaging modalities such as diffusion and perfusion MRI, MR spectroscopy, positron emission tomography (PET) or single photon emission computed tomography (SPECT) are required for additional information in TLN diagnosis [11].

#### Diffusion and perfusion MRI

Diffusion MRI detects the change in the Gaussian motion of protons in water, which allows the microscopic characterization of tissue [12]. Diffusion tensor imaging (DTI) using diffusion MR imaging principle obtains maps of fractional anisotropy, mean diffusivity and apparent diffusion coefficient (ADC). TL lesions show high diffusion on the ADC map while recurrence tumors show low signals [12, 13]. This may be caused by liquefaction and increase in water in the interstitial spaces due to cell necrosis in TLN, while the densely packed tumor cells in recurrent tumors restrict the water molecule motion [11]. Xu et al. [14] reported that the mean ADC ratio of patients with radiation injury was significantly higher than patients with recurrent tumor (1.62 vs 1.34, *p* < 0.01). Diffusion kurtosis imaging (DKI), which is a fourth-order three-dimensional tensor MRI technique, was reported to be able to quantify non-Gaussian water distribution changes for evaluating pathophysiological changes of the microstructural components in the brain including TLN [15].

Perfusion MRI detects cerebral blood flow and produces relative cerebral blood volume (rCBV) map due to transient drop in signal intensity by the magnetic susceptibility effect of contrast medium [12]. TLN has lower vascularity and therefore lower signal when compared with recurrent tumors that have high blood volume and blood flow due to neovascularization. Barajas et al. [16] reported that the mean and maximum rCBV were significantly lower in the radiation-induced necrosis group compared with recurrent metastatic tumor group. In addition, the mean, minimum and maximum percentage of signal-intensity recovery (PSR) values were significantly higher in radiation-induced necrosis cases. These studies showed that diffusion and perfusion MRI could be useful tools for differentiating TLN from recurrent tumors and DKI could be further explored for imaging of early post-RT TL changes.

#### MR spectrometry

MR spectrometry is a non-invasive diagnostic modality for the detection of metabolites in tissue in vivo. The detectable metabolites include choline (Cho), creatine (CR) and N-acetylaspartate (NAA). TLN usually has lower Cho signal when compared with closely packed tumor cells due to cell proliferation [17]. Cr represents energy metabolism with little variation while NAA represents neural integrity marker and is usually lowered in both TLN and tumor. A study by Chong et al. [18] found that there was decrease of NAA in areas of TLN while Cho levels could be more varied possibly due to oligodendrocyte injury. Another study demonstrated that during the acute phase of TLN (< 6 months post-RT), NAA/Cho ratio was significantly lower in post-RT patients compared with healthy controls while the later stage of TLN (6–12 months post-RT) showed the recovery of NAA/Cho ratio [19].

#### Positron emission tomography (PET) and single photon emission computed tomography (SPECT)

Fluorine-18 (F-18) fluorodeoxyglucose (FDG) uptake of PET appeared to be a suitable candidate for differential diagnosis of radiation-induced necrosis and recurrent tumor as they are hypometabolic and hypermetabolic respectively. Early studies conducted on differentiating the two type of lesions demonstrated encouraging performance of F-18 FDG PET [20, 21]. However, conflicting results were evaluated by Ricci et al. [22], who suggested that using PET scan results as sole determinant of therapy would have caused 32% of patients to be treated wrongly. The sensitivity and specificity were 86 and 22% respectively when contralateral white matter was employed as the reference. Meanwhile, the rates were 73 and 56% respectively when contralateral grey matter was used as the reference instead. Therefore, PET appeared to have lower differentiating ability when compared to functional MRIs.

Thallium-201 (Tl-201) chloride SPECT offers a simpler and cheaper alternative to PET. In a study comparing between SPECT and PET in the diagnosis of tumor recurrence, similar sensitivity and specificity were obtained [23]. While for the evaluation of diagnostic value of SPECT in differentiating radiation injury and tumor recurrence, another study reported that the Tl index of < 3.0 was related to radiation injury while an index of > 5.0 was related to tumor recurrence [24]. For the Tl index between 3.0 and 5.0, SPECT was repeated monthly until the index was > 5.0 or < 3.0. If the index of a lesion remained between 3.0 and 5.0 for 2 months, radiation injury was confirmed. This method achieved a sensitivity of 91%, which was higher than that of PET.

### Onset

Great variation on the TLN onset time in post-RT NPC patients has been reported. It was found to be affected by RT technique, dosage and TLN evaluation method. For patients treated by conventional RT, one study reported the mean latent period from the end of the last radiation to the diagnosis of TLN was 26.3 months using surgical intervention, CT or MRI for diagnosis [4]. Another group studied TLN based on the detection of white-matter lesions, contrast-enhanced lesions or cysts by MRI reported that the median latency was 49.8 months [7]. Other studies on IMRT-induced TLN based on MRI reported that the median latency period were 33–38 months [7, 9, 25]. Interestingly, a study found that IMRT group has significantly shorter onset time than conventional RT group (36.9 months vs 49.8 months, *p* < 0.001) [7]. In addition, another study on re-irradiation of recurrent NPC using IMRT revealed that the median latency period of TLN after the completion of second RT course was 15.0 months based on MRI data [26].

The underlying mechanisms of TLN and other radiation-induced central nervous system (CNS) damages remain poorly understood. This is because it is difficult to simulate the condition using in vitro and animal systems due to complexity of CNS and dose pattern, time course and feedback mechanisms [27]. Belka et al. [27] proposed that damage of vascular structures and oligodendrocytes could contribute to the development of CNS toxicity. An animal study has demonstrated that the disruption of the blood-brain and blood-spinal barriers may contribute to TLN [28]. This was echoed by another study conducted on radiation-induced acute blood-brain barrier disruption which reported that the increase in permeability of the blood-brain barrier by radiation could be followed by secondary radiation-induced necrosis [29]. Using MRI as the imaging modality for TLN, Wang et al. [30] suggested that TLN development started with white-matter changes, followed by contrast-enhanced lesions and finally cysts presentation in the later stage. Another study also suggested that the solid enhanced nodular lesions were the early stage of TLN development detected by MRI [31], in which the enhanced nodular lesion of < 0.8 cm did not have necrosis whereas lesions > 2.0 cm demonstrated a necrotic core.

### Predictive parameters and dose constraints

The incidence of TLN after RT varied greatly among different oncology centres. Several studies demonstrated that IMRT offered better protection of TLs than conventional 2-dimensional RT. Zhou et al. [7] reported that the actuarial 5-year incidence of TLN was significantly lower in IMRT (16%) than conventional RT (34.9%) (*p* < 0.001), in which IMRT could significantly reduce the risk of TLN in T1 – T3 diseases but not the T4 disease. This finding was further elaborated by Su et al. [8] who explained that part of TL might be delineated as target in some advanced T-stage NPC patients, causing the unavoidably high TL dose and subsequent TLN even if IMRT was used. Interestingly, another study reported that more extensive white-matter lesions and greater maximum diameter of contrast-enhanced lesions were demonstrated using MRI in the conventional RT group than the IMRT group [32].

While it is difficult to assess the relationship between TLN and actual dose volume of TLs due to technical limitations [25], QUANTEC (Qualitative Analysis of Normal Tissue Effects in the Clinic) suggested that the dose limits for TL D_max_ ≤ 60 Gy and V_65Gy_ ≤ 1% are commonly accepted in clinical use. However, QUANTEC constraints are based on past experience from the 3-dimensional conformal RT or 2-dimensional conventional RT era and may not be suitable for IMRT. Because of this, Wang et al. [33] generated a model to predict TLN occurrence using clinical dosimetric parameters using cross-validation LASSO (least absolute shrinkage and selection operator) regression method. They reported that only physical dose parameters (D_0.5cc_ and D_10_) were reliable factors for the prediction of TLN, while all clinical parameters such as age, gender, stage, diabetes and hypertension did not have direct impact on TLN. In addition, several teams have recently studied on the predictive parameters and dose constraints related to radiation induced TLN. The suggested dose constraints were commonly expressed in “dose received by a certain volume x” (e.g. D_xcc_), or “volume of the TL receiving a specified dose y Gy” (e.g. V_yGy_). A summary is shown in Table 1. Feng et al. [9] worked on the logistic regression model and showed that the TD_5/5_ (tolerance dose for TLN incidence of ≧5% in 5 years) and TD_50/5_ (tolerance dose for TLN incidence of ≧50% in 5 years) of D_2_cc (dose delivered to 2 cm^3^ volume of TL) were 60.3 Gy and 76.9 Gy respectively and suggested that the biological effective dose thresholds of D_2_cc should be considered in IMRT planning of NPC. Sun et al. [34] suggested D_0.5_cc of 69 Gy as the dose tolerance of TLs and the risk of TLN was highly dependent on high dose ‘hot spots’ of TLs. Su et al. [8] claimed that D_max_ (maximum point dose) and D_1_cc (dose delivered to 1 cm^3^ volume of TL) in TLN cases were significantly greater than the patients without TLN and advocated that IMRT with D_max_ <  68 Gy or D_1_cc <  58 Gy was relatively safe. They also showed rV_40_ (percentage volume of TL receiving ≧40 Gy) < 10% or aV_40_ (absolute volume of TL receiving ≧40 Gy) < 5 cm^3^ of TLs was effective to prevent TLN. Zeng et al. [25] showed that D_1_cc of 62.83 Gy could be the dose tolerance of the TL and they suggested TD_5/5_ and TD_50/5_ of D_1_cc were 62.8 Gy and 77.6 Gy respectively. For patients with locally advanced tumour, a reasonable balance between adequate tumour coverage and risk of TLN is needed. Huang et al. suggested a dose limit of D_1cc_ ≤ 71.14 Gy and D_max_ ≤ 72 Gy for T4 disease [35]. Based on the same rationale, Lee et al. [36] recommended a D_0.03cc_ PRV dose of ≤65 Gy for T1–2 tumours and a maximum acceptable criterion (MAC) of ≤70 Gy for T3–4 tumours. Besides the stress on controlling focal high doses for preventing TLN, Zhou et al. [37] reported that aV_45_ had the strongest predictive power for the volume of TLN lesions and aV_45_ of < 15.1 cm^3^ was proposed as the dose constraint for preventing large TLN lesions of > 5 cm^3^. In addition, Kong et al. [38] also selected D_max_ and D_1_cc as the predictors and calculated the TD_5/5_ and TD_50/5_ were 69.0 Gy and 82.1 Gy for D_max_, and 62.8 Gy and 80.9 Gy for D_1_cc respectively.

**Table 1:** Summary of previous studies on the predictive parameters and dose tolerance of radiation induced temporal lobe necrosis (TLN)

Literature	Predictive Parameter	Proposed Dose Tolerance	Sample size (n)
Feng et al., 2018 [9]	D_2_cc	TD_5/5_ = 60.3 Gy TD_50/5_ = 76.9 Gy	695
Sun et al., 2013 [34]	D_0.5_cc	69.0 Gy	20
Su et al., 2012 [8]	D_max_ D_1_cc rV_40_ aV_40_	< 68.0 Gy < 58.0 Gy < 10% 5 cm^3^	870
Zeng et al., 2015 [25]	D_1_cc	TD_5/5_ = 62.8 Gy TD_50/5_ = 77.6 Gy	351
Huang et al., 2019 [35]	D_1cc_ D_max_	≤ 71.14 Gy ≤ 72 Gy	506
Lee et al., 2019 [36]	D_0.03cc_ (T1–2 tumour) MAC (T3–4 tumour)	≤ 65 Gy ≤ 70 Gy	21^#^
Zhou et al., 2014 [37]	aV_45_	< 15.1 cm^3^	43
Kong et al., 2016 [38]	D_max_ D_1_cc	TD_5/5_ = 69.0 Gy TD_50/5_ = 82.1 Gy TD_5/5_ = 62.8 Gy TD_50/5_ = 80.9 Gy	132

D_2_cc = dose delivered to 2 cm^3^ volume of TL, Gy = Gray, TD_5/5_ = tolerance dose for TLN incidence of ≧5% in 5 years, TD_50/5_ = tolerance dose for TLN incidence of ≧50% in 5 years, D_0.5_cc = dose delivered to 0.5 cm^3^ volume of TL, D_max_ = maximum point dose, D_1_cc = dose delivered to 1 cm^3^ volume of TL, rV_40_ = percent of TLs receiving ≧40 Gy, aV_40_ = absolute volume of TLs receiving ≧40 Gy, D_0.03_cc = dose delivered to 0.03 cm^3^ volume of TL, MAC = maximum acceptable criteria, aV_45_ = absolute volume of TLs receiving ≧45 Gy. ^#^number of oncology centres

Apart from the dose-volume parameters, dose scheme (fractionation) and re-treatment were also identified as predictive factors for TLN development. For the impact of dose scheme on TLN, Lee et al. [6] commented that an increase in dose per fraction could significantly increase the incidence rate of TLN despite a lower total dose. The same team also suggested the decrease in overall treatment time could significantly increase the risk of TLN (Hazard ratio 0.88, 95% Confidence interval 0.80–0.97) [39]. On the other hand, another group studied on hyperfractionation schedule of 67.2 Gy in 42 fractions in 6 weeks found that it significantly increased TLN incidence [40]. For the re-irradiation of locally recurrent NPC, Liu et al. [26] demonstrated that the interval time between the two courses of RT and the summation of the maximum doses of the two RT courses were the independent factors of TLN incidence. The study suggested a minimum of 2-year interval time between two RT courses and limiting the total maximum dose of the two RT courses to 125 Gy as directions to reduce TLN incidence.

Recently, artificial intelligence such as radiomics has been introduced to predict treatment effect including complications and disease progression. Radiomics describes a broad set of computational methods that extract quantitative features from radiographic images. The resulting features can be used to inform imaging diagnosis, prognosis, therapy response and radiation-related toxicity in oncology [41]. Its applications on head and cancers have been increasing [42, 43]. For instance, Zhou et al. used radiomics to predict treatment outcome and toxicities of brain tumours [44]. In addition, Peng et al. built a predictive model on radiomic features for radiation induced complication and disease progression in brain lesions treated with stereotactic radiotherapy [45]. Such technology has the potential to be used for prediction of TLN in post-RT NPC patients.

### Management

#### Surgery

To the present knowledge, there is no effective treatment to reverse or stop the development of late stage cerebral radiation-induced necrosis including TLN. Since severe TLN patients can experience potentially fatal symptoms including increase in intracranial pressure, surgery remains to be the treatment of choice [46]. Temporal lobectomy with the excision of the necrotic tissue and abscess cavity was commonly conducted for patients with radiation induced TLN [47]. An earlier report of treatment outcomes following surgery for TLN patients revealed that there were symptomatic recurrence developed in post-operative TLN patients [48], which led to severe debilitation and death. However, with the advancement in surgical techniques, the outcome of surgery has been improved in recent studies. Fang et al. [46] evaluated the mean post-operative follow-up period of 29.8 months in 16 late stage TLN patients receiving surgery. Only 1 patient experienced recurrence of TLN and required another operation while other patients had good recovery. Another group also evaluated a similar mean post-operative follow-up period of 29 months in a series of 14 TLN patients [49]. Apart from good recovery, this group also commented that surgery was useful for establishing accurate diagnosis.

#### Steroid

Steroid treatment has been established as the mainstay of treatment for TLN patients with mild or no symptoms. The common regimen is oral dexamethasone 4 to 16 mg/day for 4 to 6 weeks and is gradually tapered off in 4 months [4, 5, 48]. Pulse steroid of methylprednisolone by intravenous infusion over 3 consecutive days with 1 g administration and then followed by administration of oral prednisolone for 10 days tail off has also been used in recent protocols [5]. The efficacy of steroid treatment varied in different studies. In an earlier study, Lee et al. [48] reported that 19.4 and 15.3% of 72 TLN NPC patients could achieve complete response and partial response respectively after oral dexamethasone treatment. Another study produced similar favorable outcome of steroid treatment when applied to 7 NPC patients with post-RT cerebral radiation-induced necrosis [4]. A recent study that compared the outcomes of conventional oral steroid, pulse steroid and conservative treatment in 174 NPC patients with mild or asymptomatic TLN reported that pulse steroid therapy was associated with higher clinical response rate than conventional oral steroid. They also found that the use of steroid treatment did not affect complication-free survival. Nevertheless, steroid treatment has been suggested to be associated with fatal sepsis due to induced immune-suppression, though no definite conclusion has been reached yet [5, 48].

#### Hyperbaric oxygen treatment (HBOT)

HBOT aims to improve tissue oxygen concentration for promoting angiogenesis to potentially heal radiation-induced necrosis. HBOT consisted of 20 or more treatment sessions at 2.0 to 2.4 atm with 100% oxygen inhalation for 1.5 to 2.0 h [50]. This treatment was claimed to be useful for initial improvement or stabilization of symptoms in radiation-induced necrosis caused by a range of children cerebral cancers [50]. Another study that applied HBOT to a 68-year-old male patient presented with radiation-induced brain necrosis after stereotactic radiosurgery reported improvement in clinical conditions [51]. However, to date, there has been no specific study conducted for assessing the effectiveness of HBOT in TLN of NPC patients and therefore the clinical potential of HBOT has yet to be confirmed.

#### Bevacizumab

Bevacizumab is initially a targeted therapy drug for some cancers by inhibiting the angiogenesis factor vascular endothelial growth factor (VEGF). Recently, this drug has been investigated for treating cerebral radiation-induced necrosis including TLN. In a case study of a NPC patient who developed post-RT TLN treated with 4 doses of 5 mg/kg of bevacizumab every other week, it was reported that there was relief of clinical symptoms up to 6 months follow up [52]. There are other clinical studies suggesting the effectiveness of bevacizumab in radiation-induced necrosis after poor treatment outcome of steroids [53–55]. A randomized control trial by Levin et al. [56] on 14 patients with radiation-induced necrosis concluded that there was Class I evidence of bevacizumab efficacy for treating cerebral radiation-induced necrosis after a median follow up of 10 months. Li et al. [57] administered bevacizumab in 50 patients with post-RT TLN found that the median percentage of decrease in TLN volume assessed by T2-weighted MRI was 72.6%, in which 38 (76%) patients demonstrated effective response. In addition, they reported that D_max_ of temporal lobe greater than 75.5 Gy would reduce the effectiveness of bevacizumab. All these reports suggested that bevacizumab could be a promising treatment strategy apart from surgery and steroid treatment.

## Conclusion

TLN of radiation-treated NPC patients could be a devastating late complication. Moreover, the differential diagnosis of TLN remains challenging by conventional imaging and may require the additional use of advanced imaging modalities. Although IMRT could effectively reduce the incidence of TLN in T1 – T3 patients, the incidence of TLN in T4 patients is still significant and therefore requires regular follow-up. Various dose constraints expressed in terms of dose-volume parameters have been suggested for the prevention of TLN development, and predictive models involving dosimetric parameters have been developed. Meanwhile, the complex relationship between TL dose parameters and TLN incidence warrants large-scale longitudinal studies and it is expected that the alternation of dose scheme can influence incidence rate of post-RT TLN. Although there is no definitive treatment for TLN, bevacizumab has emerged to be an encouraging treatment option other than the long-established surgery and steroid treatment.

## Data Availability

Not applicable.

## Abbreviations

ADC: Apparent diffusion coefficient
aV_40_: Absolute volume of TL receiving ≧40 Gy
aV_45_: Absolute volume of TL receiving ≧45 Gy
Cho: Choline
CNS: Central nervous system
CR: Creatine
CT: Computed tomography
CTV: Clinical target voluem
D_0.5_cc: Dose delivered to 0.5 cm^3^ volume of TL
D_0.03_cc: Dose delivered to 0.03 cm^3^ volume of TL
D_1_cc: Dose delivered to 1 cm^3^ volume of TL
D_2_cc: Dose delivered to 2 cm^3^ volume of TL
DKI: Diffusion kurtosis imaging
D_max_: Maximum dose
DTI: Diffusion tensor imaging
FDG: Fluorodeoxyglucose
GTV: Gross tumour volume
Gy: Gray
HBOT: Hyperbaric oxygen treatment
IMRT: Intensity modulated radiotherapy
LASSO: least absolute shrinkage and selection operator
MAC: Maximum acceptable criteria
MRI: Magnetic resonance imaging
NAA: N-acetylaspartate
NPC: Nasopharyngeal carcinoma
PET: Positron emission tomography
PSR: percentage of signal-intensity recovery
rCBV: relative cerebral blood volume
QUANTEC: Qualitative Analysis of Normal Tissue Effects in the Clinic
RT: Radiotherapy
rV_40_: Percentage volume of TL receiving ≧40 Gy
SPECT: Single photon emission computed tomography
TD_5/5_: Tolerance dose for TLN incidence of ≧5% in 5 years
TD_50/5_: Tolerance dose for TLN incidence of ≧50% in 5 years
TLN: Temporal lobe necrosis
VEGF: Vascular endothelial growth factor
